# Serotonin in Cardiovascular Disease: From Pathophysiological Insights to Clinical Utility

**DOI:** 10.3390/diagnostics16132068

**Published:** 2026-07-01

**Authors:** Aleyma Veliz Perez, Mihaela Badea, Elena Laura Gaman

**Affiliations:** 1 Department of Fundamental, Prophylactic and Clinical Disciplines, Faculty of Medicine, Transilvania University of Brasov, 56 Nicolae Balcescu, 500019 Brasov, Romania; aleyma.veliz@unitbv.ro; 2 Research Center for Fundamental Research and Prevention Strategies in Medicine, Research and Development Institute, Transilvania University of Brasov, 10 Institutului St., 500484 Brasov, Romania; 3 Faculty of Medicine, Carol Davila University of Medicine and Pharmacy, Bdul Eroilor Sanitari 8, 050474 Bucharest, Romania; gaman.laura@umfcd.ro

**Keywords:** serotonin, coronary heart disease, cardiac biomarkers, microvascular dysfunction, precision medicine, atherosclerosis

## Abstract

Cardiovascular disease (CVD) remains the leading cause of morbidity and mortality worldwide, posing a significant and ongoing burden on healthcare systems. Early identification of reliable biomarkers is essential for improving risk stratification, diagnosis, and therapeutic monitoring. Serotonin (5-hydroxytryptamine, 5-HT) has recently emerged as a candidate biomarker implicated in early cardiovascular pathophysiological processes, including platelet activation, vascular smooth muscle cell dysfunction, endothelial impairment, and intraplaque inflammation. This narrative review synthesizes current evidence from peer-reviewed literature retrieved from major scientific databases, including MDPI, PubMed, Scopus, Web of Science, and Elsevier. Relevant studies were selected for their contributions to understanding the role of serotonin in cardiovascular physiology and pathology, with a focus on its potential utility as a biomarker. Serotonin exerts pleiotropic effects within the cardiovascular system, extending beyond its established role as a neurotransmitter. It plays a critical role in platelet aggregation, regulation of vascular tone, and vascular remodeling. Experimental and translational studies suggest that altered serotonin signaling is associated with endothelial dysfunction, atherogenesis, and thrombotic processes. These findings support that serotonin may have potential as a novel or adjunctive biomarker in cardiovascular disease. Although emerging evidence highlights the relevance of serotonin in cardiovascular pathophysiology, its clinical utility as a biomarker remains limited by insufficient large-scale clinical validation and lack of standardized measurement approaches. At present, serotonin should be considered an investigational biomarker. Further well-designed prospective studies are required to establish its diagnostic and prognostic value and to determine its applicability in routine clinical practice.

## 1. Introduction

Cardiovascular disease (CVD) remains the leading cause of morbidity and mortality worldwide, accounting for approximately 17.9 million deaths annually, as reported by the World Health Organization [1]. Despite sustained global efforts and substantial investments by international organizations, governments, and research institutions to improve prevention, early diagnosis, and therapeutic strategies, CVD continues to impose a major burden on healthcare systems and is responsible for nearly one-third of all deaths globally [1]. These data underscore the persistent need for improved diagnostic and prognostic tools capable of identifying disease at earlier and potentially reversible stages.

In this context, biomarkers have emerged as essential components in the clinical management of cardiovascular diseases, contributing to diagnosis, risk stratification, and therapeutic monitoring. Biomarkers provide objective and quantifiable indicators of underlying pathophysiological processes that are often not detectable through clinical assessment alone, thereby enabling more accurate evaluation of disease progression and patient outcomes [2]. While established biomarkers, such as cardiac troponins, reflect myocardial injury and necrosis, there is growing interest in identifying emerging biomarkers that capture earlier, upstream mechanisms underlying CVD pathogenesis.

Serotonin (5-hydroxytryptamine, 5-HT) was first described in the mid-20th century as a vasoactive compound present in blood serum capable of inducing vasoconstriction, establishing its role as a biologically active mediator in the cardiovascular system prior to its recognition as a central neurotransmitter [3]. In the peripheral circulation, serotonin is not freely dissolved in plasma but is actively taken up by platelets via the serotonin transporter (SERT) and stored in dense granules. Upon platelet activation, serotonin is released into the local microenvironment, where it contributes to platelet aggregation, vasoconstriction, and thrombus formation. This mechanistic link highlights the central role of serotonin in platelet-driven thrombosis and supports its candidacy as a biomarker of thrombotic cardiovascular risk [4].

In addition to its role in hemostasis, serotonin is synthesized and metabolized within cardiac tissues, where it exerts autocrine and paracrine effects through specific receptor subtypes, particularly 5-HT_4_ receptors. Activation of these receptors modulates myocardial contractility, influences cardiac rhythm, and contributes to structural remodeling processes [5].

Serotonin is widely recognized as a central neurotransmitter involved in autonomic cardiovascular regulation through sympathetic and parasympathetic pathways. Synthesized predominantly by intestinal enterochromaffin cells and sequestered by circulating platelets, peripheral serotonin does not cross the blood–brain barrier and operates through distinct biosynthetic, transport, and receptor mechanisms [5,6]. This review focuses on this peripheral serotonergic system and its direct cardiovascular effects. Increasing evidence indicates that dysregulation of serotonergic signaling is associated with pathological conditions such as heart failure, arrhythmogenesis, endothelial dysfunction, and atherosclerosis [5].

Collectively, serotonin may be considered as a potential emerging biomarker reflecting early-stage cardiovascular alterations, including platelet activation, vascular smooth muscle cell dysfunction, and intraplaque inflammation. Importantly, serotonin may provide complementary pathophysiological information to established necrosis-associated biomarkers, such as cardiac troponins, by capturing upstream functional and inflammatory processes within the coronary vasculature [6].

Recently, numerous biomarkers have been identified. The use of risk markers has transformed cardiovascular medicine, exemplified by the routine assessment of troponin for both diagnosis and prognosis in patients with chest pain. Clinical risk factors, together with biochemical, cellular, and imaging parameters, provide a more comprehensive perspective. Identifying novel risk factors may enable improved risk stratification and a steady, gradual progression toward precision medicine. While these traditional markers are essential for risk stratification, serotonin may provide complementary information for a multidimensional perspective on atherosclerosis and microvascular dysfunction [7].

This narrative review synthesizes the evidence from population-based studies, clinical trials, and communication research to evaluate the predictive value, limitations, and potential of serotonin as a novel cardiac biomarker to describe its potential as an emerging cardiac biomarker for the diagnosis, prognosis, and pathophysiology of cardiovascular diseases, the leading cause of global morbidity and mortality.

## 2. Materials and Methods

This narrative review was conducted to evaluate the potential role of serotonin (5-hydroxytryptamine, 5-HT) as a potential emerging cardiac biomarker. A comprehensive literature search was performed using the following electronic databases: MDPI, PubMed and Scopus. Publisher platforms: MDPI and Elsevier) were not used as primary databases but were accessed via the abovementioned databases, to ensure broad coverage of relevant peer-reviewed studies.

The search strategy combined the following keywords using Boolean operators (“serotonin” OR “5-hydroxytryptamine” OR “5-HT”) AND (“cardiovascular disease” OR “atherosclerosis” OR “coronary microvascular dysfunction” OR “myocardial infarction” OR “platelet activation” OR “unstable angina”). Additional relevant articles were identified through manual screening of reference lists from selected publications. The literature search was conducted between January and February 2026. No restrictions were imposed on the publication date to capture both foundational and recent evidence. Studies were considered eligible if they addressed the role of serotonin in cardiovascular physiology or pathology, including its involvement in platelet function, vascular biology, myocardial processes, and atherosclerosis.

Inclusion criteria: original research or peer-reviewed reviews addressing serotonin in cardiovascular physiology or pathology; studies reporting quantitative data on serotonin levels in relation to cardiovascular outcomes; human or animal studies with clear methodological descriptions.

Exclusion criteria: studies focused exclusively on central nervous system effects of serotonin without cardiovascular data; non-English articles.

A total of 192 articles were initially identified. After the removal of duplicate records (*n* = 89), 103 titles and abstracts were screened. Of these, 58 were excluded based on relevance criteria. The remaining 45 full-text articles were assessed for eligibility, and 32 were included in this narrative review.

Given the narrative nature of this review, no formal meta-analysis or quantitative synthesis was performed. Instead, studies were qualitatively assessed and integrated to provide a comprehensive overview of current knowledge and emerging evidence regarding serotonin as a cardiovascular biomarker.

## 3. Serotonin in Cardiovascular Disease: A Multifaceted Modulator Beyond Platelet Aggregation

Serotonin contributes to several cardiovascular processes: platelet aggregation and thrombosis, vasoconstriction and regulation of vascular tone, smooth muscle proliferation, cardiac remodeling, and fibrosis [8].

Enterochromaffin cells in the intestinal mucosa produce approximately 95% of the total 5-HT in the human body. Serotonin is synthesized by tryptophan hydroxylase 1 (TPH1) and is stored in platelets [9]. Then it is released into the blood and rapidly sequestered by circulating platelets via SERT [5].

The enzyme tryptophan hydroxylase 1 (TPH1), expressed in enterochromaffin cells, catalyzes the rate-limiting reaction in peripheral serotonin biosynthesis, and it represents a mechanistically different compartment from central serotonin synthesis, which is mediated by TPH2 in serotonergic neurons of the raphe nuclei. This peripheral biosynthetic axis exerts systemic metabolic regulation. Crane et al. demonstrated in Tph1^−/−^ mice that gut-derived peripheral serotonin suppresses brown adipose tissue (BAT) thermogenesis by blunting β-adrenergic induction of the thermogenic program in brown and beige adipocytes, promoting obesity, insulin resistance, and hepatic steatosis. Pharmacological TPH1 inhibition in wild-type mice recapitulated these protective metabolic effects, confirming causality and establishing the role of peripheral serotonin as a systemic regulator of cardiometabolic homeostasis [10].

Notably, peripheral serotonin does not cross the blood–brain barrier (BBB). Platelets serve as the primary reservoir for 5-HT in the periphery, storing it in dense granules and releasing it upon activation at sites of endothelial injury or within the microenvironment of a developing atherosclerotic plaque [4].

5-HT exerts its effects on the vascular wall through an array of receptor subtypes, most notably 5-HT2A and 5-HT2B. The 5-HT2A receptor is abundantly expressed in vascular smooth muscle cells (VSMCs) and platelets. Activation of 5-HT_2_A initiates a cascade that promotes the proliferation and migration of VSMCs from the media into the intima, thereby driving intimal thickening and plaque development [8,9,11].

5-HT is not only a result of platelet activation; it is also involved in angiogenesis, in which endothelial cells (ECs) play a principal role. Stimulation of 5-HT1 and/or 5-HT2 receptors has been implicated in 5-HT’s angiogenic effects. The extracellular signal-regulated kinase and endothelial nitric oxide (NO) synthase (eNOS) activation-dependent pathways are involved in the mechanisms [8]. One of the most important roles of 5-HT in Endothelial Cells (ECs) is to activate endothelial NOS (eNOS). Indeed, 5-HT is a dominant substance released by aggregating platelets and triggers NO production. NO plays a pivotal role in the endothelium’s protective function against coronary disease [10].

Sadykova et al. [11] investigated the expression and functional significance of serotonin, its membrane transporter, and 5-HT2A/5-HT2B receptors in the development of early atherosclerotic changes in low-density lipoprotein receptor-deficient (Ldlr^+/−^) mice. The study demonstrated a statistically significant increase in the expression of these targets in gene-modified mice, supporting their mechanistic involvement in atherosclerotic plaque formation [11] (Table 1).

Sadykova et al. [12], in a cross-sectional study involving 116 pediatric patients with familial hypercholesterolemia, performed a comprehensive evaluation of the serotonergic system by quantifying plasma and platelet levels of serotonin (5-HT), its primary metabolite 5-hydroxyindoleacetic acid (5-HIAA), and the expression of the serotonin transporter (SERT) in platelets. These parameters were subsequently correlated with established indices of arterial stiffness, including pulse wave velocity (PWV). The authors reported a significant positive association between serotonergic markers and vascular stiffness, suggesting that alterations in serotonin signaling may occur at early, subclinical stages of vascular dysfunction, preceding the development of overt atherosclerotic disease.

Complementary evidence is provided by studies conducted in 2024 and 2025 by Sadykova and colleagues [11,12], which further investigated the role of the membrane serotonin transporter (SERT) in the context of hypercholesterolemia. In addition to pediatric clinical cohorts, these investigations incorporated experimental data derived from immature heterozygous LDL receptor-deficient (Ldlr^+/−^) mice, a well-established model of familial hypercholesterolemia. These studies demonstrated altered expression patterns of SERT and serotonin receptors, particularly 5-HT_2_A and 5-HT_2_B, in the aortic wall and left ventricular myocardium, implicating serotonergic signaling in early vascular and myocardial remodeling processes.

Notably, both the clinical and experimental studies focus on early, subclinical stages of disease—namely, asymptomatic children with familial hypercholesterolemia and immature animal models exhibiting pre-atherosclerotic lesions. This convergence of evidence supports the concept that serotonin is not merely a vasoactive mediator but a biologically relevant modulator of atherogenesis, contributing to both the initiation and progression of vascular pathology.

Table 2 compiles genetic and pharmacological studies cited in this review that have investigated the role of serotonin in atherosclerosis and vascular dysfunction.

Table 2 summarizes the experimental models, both animal and ex vivo. These studies provide evidence that peripheral serotonin promotes atherogenesis through 5-HT2A receptor-mediated mechanisms, including VSMC contraction, platelet aggregation, and macrophage infiltration. The ex vivo studies with 5-HT2A antagonists show that pharmacological blockade of this receptor abolishes serotonin-induced vasoconstriction in rat aortic rings and inhibits platelet aggregation in human platelet-rich plasma induced by serotonin, ADP, and collagen [14]. These findings confirm that 5-HT2A antagonism exerts protective cardiovascular effects by preventing both vascular smooth muscle contraction and thrombus formation, providing a strong rationale for further investigation of serotonin as an exploratory biomarker of vascular dysfunction and atherosclerotic disease progression. Its potential role in identifying plaque vulnerability and instability warrants detailed exploration, as discussed in the following section.

## 4. Serotonin: From Vascular Inflammation to Thrombotic Threat—A Key Mediator of Vascular Pathology

Serotonin acts as a growth factor, stimulating the proliferation and migration of vascular smooth muscle cells and promoting atherosclerosis [15,16]. Serotonin works in concert with cytokines such as Tumor Necrosis Factor-α (TNF-α) and Interleukin-6 (IL-6) to maintain a state of chronic, non-resolving inflammation [13].

The relationship between serotonin and oxidative stress has been investigated in metabolic contexts; Bianchi et al. [14] demonstrated that serotonin, degraded by mitochondrial monoamine oxidase A (MAO-A) in cardiomyocytes, generates hydrogen peroxide (H_2_O_2_), leading to intracellular oxidative stress, NF-κB activation, and proinflammatory cytokine production; independently of membrane serotonin receptors and contributes to post-ischemic myocardial injury. In this study, MAO-A inhibition significantly reduced infarct size in rat hearts exposed to ischemia–reperfusion [14].

Mitochondrial ROS (mtROS) production serves as a unifying pathogenic trigger, promoting endothelial dysfunction, vascular inflammation, and cellular apoptosis. mtROS induces endothelial dysfunction through three key mechanisms: direct scavenging of nitric oxide (NO), uncoupling of endothelial nitric oxide synthase (eNOS), and subsequent impairment of vasomotion [17,18].

**Figure 1:**
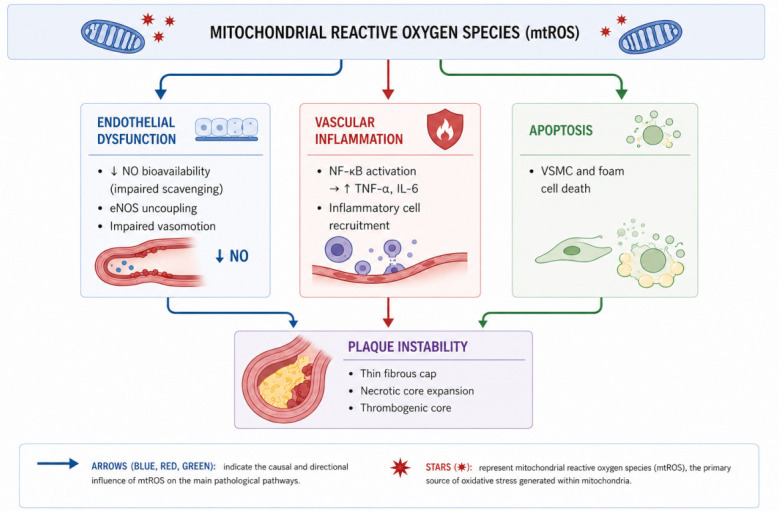
Mitochondrial ROS as a central driver of atherosclerotic plaque instability. Mitochondrial ROS (mtROS) drives endothelial dysfunction (via NO scavenging and eNOS uncoupling), vascular inflammation (via NF-κB activation and cytokine release), and apoptosis of vascular cells, leading to necrotic core expansion, fibrous cap thinning, and increased plaque vulnerability. Adapted from Münzel et al. [17].

This dysfunctional endothelium, driven by mtROS, becomes activated, promoting the recruitment of inflammatory cells and leading to vascular inflammation. Sustained oxidative stress also induces cellular damage within the vessel wall, leading to cell death. As reviewed by Münzel et al. [17], mtROS can trigger both apoptosis (programmed cell death) and necrosis. This critically influences the formation of the necrotic core, which results from the accumulation of apoptotic lipid-laden macrophage foam cells (cellular debris and lipids), forming a soft, acellular, and highly thrombogenic core. In addition, mtROS induces apoptosis of vascular smooth muscle cells (VSMCs) within the fibrous cap. These cells are responsible for synthesizing and maintaining the collagen-rich extracellular matrix that provides the plaque with its strength and stability. Their loss leads to thinning of the fibrous cap, further compromising plaque integrity. All these processes are fundamental to atherosclerotic plaque initiation, progression, formation, and instability [17] (Figure 1).

As illustrated in Figure 1, mitochondrial ROS (mtROS) acts as a central driver linking endothelial dysfunction, vascular inflammation, and cellular apoptosis. These interconnected pathways converge to promote atherosclerotic plaque destabilization, thereby accelerating disease progression and increasing the risk of plaque rupture and thrombotic events [17].

The role of serotonin in cardiovascular regulation and thrombosis was further demonstrated in the experimental study by Marcinkowska et al. (2022) [13], who investigated the pharmacological effects of selective serotonin 5-HT_2_A receptor antagonists on platelet activity and vascular function. Using isolated rat aortic rings, the researchers examined the contractile response to serotonin and evaluated how blockade of 5-HT_2_A receptors modifies it. The most potent compound, AZ928, exhibited a p*K*__B_ value of 9.502 ± 0.09, indicating a very high antagonistic affinity that slightly exceeds that of the reference drug, ketanserin (p*K*__B_ = 9.439 ± 0.09).

Complementary platelet aggregation assays demonstrated that serotonin significantly enhances platelet activation when combined with classical agonists, highlighting its role as an amplifier of platelet aggregation during thrombus formation. Human platelet-rich plasma was used to evaluate the inhibition of platelet aggregation induced by serotonin (5-HT), ADP, and collagen. These agonists mimic physiological pathways of thrombus formation. Aggregation was measured using impedance aggregometry and expressed as area under the curve (AUC). The antiplatelet drugs ketanserin (a classic 5-HT_2_A antagonist) and sarpogrelate (a clinical antiplatelet drug) strongly reduced or abolished serotonin-induced contraction, confirming that vasoconstriction is mediated by 5-HT_2_A receptors on vascular smooth muscle cells [13]. The results of this study clearly indicate that serotonin released from activated platelets amplifies platelet aggregation and induces vascular smooth muscle contraction, particularly after endothelial injury, effects that are suppressed by 5-HT_2_A receptor blockade. Serotonin acts as a potent vasoconstrictor, especially following endothelial damage from arterial injury or atherosclerosis, by stimulating vascular smooth muscle cells via 5-HT2A receptors. When the endothelium is damaged, the contractile response to serotonin is markedly increased [13].

Also, in Vivo genetic evidence supports the causal role of platelet-derived serotonin in acute myocardial injury. Mauler et al. (2019) [19] demonstrated in Tph1^−/−^ mice (a genetic model devoid of peripheral serotonin) that platelet serotonin induces myocardial ischemia/reperfusion (I/R) injury, identifying serotonin as a potent therapeutic target in neutrophil-dependent thromboinflammation during myocardial reperfusion injury. These changes result in enhanced inflammation in the infarct area and reduced myocardial salvage, with Tph1^−/−^ mice exhibiting a ~35% reduction in infarct size. In patients with acute coronary syndrome, plasmatic serotonin levels correlated with CD11b expression on neutrophils and myeloperoxidase plasma levels. Notably, serotonin reuptake inhibition, which depletes platelet serotonin stores, reduced neutrophil degranulation and preserved cardiac function in both mice and patients on serotonin reuptake inhibitors [19].

Platelet-released serotonin activates 5-HT_2_A receptors on neutrophils, triggering neutrophil degranulation with the release of myeloperoxidase and hydrogen peroxide (H_2_O_2_), and increases surface expression of the adhesion molecule CD11b at the site of ischemic injury, which results in enhanced inflammation in the infarct area. Tph1^−/−^ mice exhibited a ~35% reduction in infarct size. The study results showed that serotonin reuptake inhibition, which depletes platelet serotonin stores, reduced neutrophil degranulation and preserved cardiac function in both mice and patients on serotonin reuptake inhibitors. These findings identify serotonin as a possible therapeutic target in neutrophil-dependent thromboinflammation during myocardial reperfusion injury, reinforcing the translational relevance of serotonin as both a mediator and a possible therapeutic target in the context of acute coronary syndromes [19].

The converging evidence from these studies illuminates a multifaceted role for the serotonin (5-HT) system in cardiovascular pathology, operating through both direct vascular mechanisms and systemic metabolic disturbances. The interaction between activated platelets and serotonin (5-HT) stimulates vasoconstriction, amplifies thrombogenic responses, and exacerbates endothelial dysfunction within atherosclerotic vessels. This is fundamental to the development of thrombogenic vasospasm and may precipitate acute coronary events, particularly in the presence of vulnerable plaques [13,17].

### 4.1. The Dual Role of Serotonin in Hypertension

Beyond its role in plaque instability, serotonin also contributes to the regulation of systemic blood pressure through receptor-dependent mechanisms. Peripheral serotonin exerts a triphasic effect on blood pressure: an initial vasodepressor phase via 5-HT_3_ receptor activation on vagal afferents, followed by a vasopressor phase mediated by 5-HT_2_A receptor stimulation on vascular smooth muscle cells (inducing vasoconstriction), and a late vasodepressor phase through 5-HT_7_ receptor activation. In hypertension, vascular responsiveness to serotonin is markedly enhanced, with evidence suggesting a receptor subtype shift from 5-HT_2_A toward 5-HT_2_B in conduit arteries, amplifying vasoconstrictor responses [4,8,13]. The clinical relevance of these findings lies in recognizing serotonergic dysregulation as a key mechanism in the pathophysiology of arterial hypertension, opening a new horizon for antihypertensive therapies based on serotonergic system modulation.

It has been demonstrated that patients with essential hypertension present elevated plasma free serotonin levels and increased platelet serotonin release compared to controls (normotensive), whereas platelet serotonin content is reduced, suggesting increased turnover. Central serotonergic pathways further modulate sympathetic outflow: activation of 5-HT1A receptors lowers blood pressure, while 5-HT2 receptor activation increases pressor responses. Clinical relevance is supported by the antihypertensive effects of 5-HT2A antagonists such as ketanserin. Emerging evidence also links gut-derived serotonin and microbiota to hypertension through modulation of vascular tone and vagal sensory pathways. Together, these findings suggest that the serotonin system is involved in essential hypertension, although this topic remains less explored than atherothrombotic disease [8,13,18].

### 4.2. Serotonin and Immune Modulation

The elevated serotonin levels have been found at sites of inflammation and thrombosis. Serotonin receptors are expressed on multiple immune cell types, including monocytes, macrophages, dendritic cells, and T lymphocytes. Through receptor-dependent signaling, the serotonergic system modulates immune cell activation, migration, cytokine production, antigen presentation, and inflammatory polarization. These mechanisms are highly relevant to cardiovascular disease, where persistent vascular inflammation contributes to endothelial dysfunction, atherosclerotic plaque development, and plaque progression. Specifically, serotonin promotes M1 macrophage polarization while suppressing the anti-inflammatory M2 phenotype, sustains NLRP3 inflammasome activation in plaque macrophages, and enhances neutrophil recruitment and T-cell activation within atherosclerotic lesions [9,16,18].

The effect of serotonin on immune cells is not only receptor-dependent, but also context-sensitive: while its overall role in atherogenesis is predominantly pro-inflammatory, serotonin also has been shown to exert anti-atherogenic effects in mast cells by reducing their adhesion to vascular walls [9,16,18].

These immunomodulatory actions additionally link serotonin to chronic vascular inflammation and plaque progression and highlight the need to consider its immune-mediated actions alongside its direct vascular and platelet effects [9,16,19].

## 5. Serotonin in Microvascular Dysfunction

It has been reported that up to 40% of patients undergoing diagnostic coronary angiography for chest pain have no significant coronary stenosis. This reveals that a substantial proportion of symptomatic patients do not have obstructive coronary artery disease (CAD) but may, instead, have non-obstructive CAD or microvascular dysfunction, or other causes of chest pain [20,21].

Contrary to what was thought years ago, nonobstructive CAD is not benign; patients with extensive nonobstructive disease or microvascular dysfunction remain at increased risk for adverse cardiovascular events, including myocardial infarction and cardiac death, and require targeted preventive therapy. The high prevalence of nonobstructive findings emphasizes the need for improved diagnostic strategies beyond conventional angiography [22].

Myocardial ischemia may be caused by different types of functional disorders involving epicardial coronary arteries, coronary microcirculation, or both. Coronary microvascular dysfunction (CMD) has emerged as a potential mechanism of myocardial ischemia (with non-obstructive coronary disease) in addition to coronary atherosclerotic disease and epicardial coronary spasm. Notably, plasma serotonin concentration is significantly higher in patients with coronary artery disease than in those without [21,22].

In a clinical study by Odaka et al. (2016) [23], 198 patients presenting with suspected angina and non-obstructive coronary arteries were evaluated, with both intracoronary acetylcholine (ACh) provocation testing and plasma serotonin levels assessed. Intracoronary ACh testing is a well-established diagnostic modality for assessing coronary vasomotor disorders. Under physiological conditions, ACh induces endothelium-dependent vasodilation mediated by nitric oxide release. In contrast, in the presence of endothelial dysfunction or coronary microvascular impairment, ACh paradoxically elicits vasoconstriction and may precipitate microvascular spasm [23].

In this study, coronary microvascular dysfunction (CMD) was defined as the presence of myocardial lactate production—reflecting ischemia—in the absence of angiographically evident epicardial coronary spasm during ACh provocation [23]. This definition enables the identification of functional microvascular abnormalities independent of obstructive coronary artery disease.

**Figure 2:**
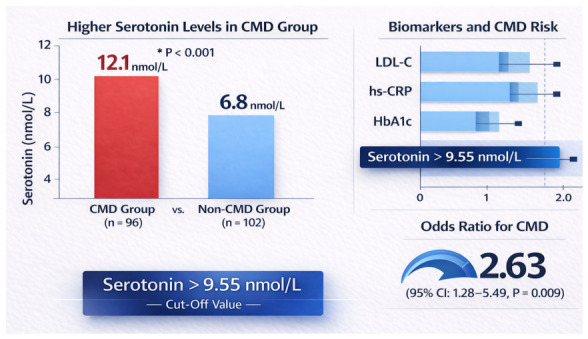
Plasma Serotonin Levels and Coronary Microvascular Dysfunction (Data from [23]). * *p* < 0.001 (CMD vs. non-CMD).

The authors demonstrated that patients with CMD had significantly higher plasma serotonin concentrations than those without CMD. Notably, when evaluated alongside conventional biomarkers, plasma serotonin emerged as the most robust independent predictor of CMD. A threshold value of 9.55 nmol/L was identified, above which serotonin levels were strongly associated with the presence of CMD. Specifically, serotonin concentrations exceeding this cut-off demonstrated the highest predictive value, with an odds ratio of 2.63 (95% confidence interval: 1.28–5.49; *p* = 0.009) [23] (Figure 2).

These findings highlight the potential utility of serotonin as a sensitive biomarker reflecting coronary microvascular dysfunction, thereby providing insight into early, functionally mediated ischemic processes that are not detectable through conventional structural assessment.

These findings identified serotonin as the first discriminator to stratify risk for CMD and positioned plasma serotonin as a promising functional biomarker for identifying patients with coronary microvascular dysfunction, particularly in those who remain undiagnosed after conventional coronary angiography.

## 6. Serotonin as a Predictor of Plaque Instability

Plaque instability is a critical determinant of acute coronary events, driven by processes such as intraplaque angiogenesis, inflammation, and extracellular matrix degradation. A vulnerable plaque is typically characterized by a large lipid-rich necrotic core (LRNC) and a thin fibrous cap (TCFA), often accompanied by high concentrations of inflammatory cells and extensive neovascularization [24,25].

Neovascularization is implicated as a key contributor to the transition from an asymptomatic fibroatheromatous plaque to a lesion vulnerable to rupture [25]. This is because these immature new microvessels are inherently fragile and prone to leakage, leading to intraplaque hemorrhage (IPH) [24,25]. The inflammatory microenvironment, mediated in part by recruited monocytes, further promotes both angiogenesis and plaque progression [26]. Serotonin contributes to this process by acting as a mitogen for endothelial cells and promoting the release of growth factors, including VEGF [26]. This mitogenic effect, mediated by the 5-HT_2_ receptor, drives the formation of disorganized neovessels that characterize vulnerable plaques [27]. These plaques are prone to rupture and thrombosis. Vulnerable plaques are responsible for 60–70% of acute myocardial infarctions [25,28].

**Figure 3:**
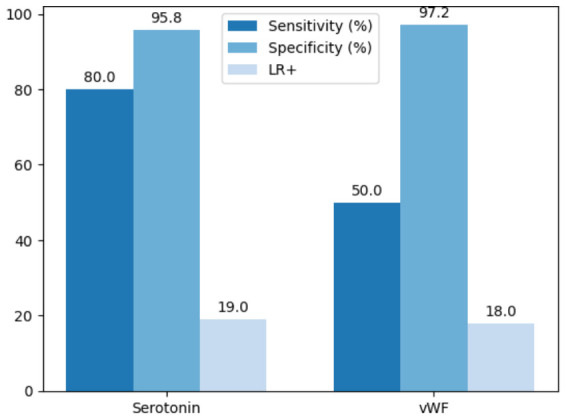
Diagnostic performance of plasma serotonin and von Willebrand factor (vWF) for predicting short-term progression from unstable angina (UA) to myocardial infarction (MI). Serotonin cut-off: >21.575 μg/mL (Se = 80.0%, Sp = 95.8%, AUC = 0.975); vWF cut-off: <0.114 rel.units/mL. (Data from Tyravska et al., 2021 [29]).

Research by Tyravska et al. (2021) [29] included 103 patients in an observational cohort study to investigate whether blood plasma serotonin content by ion-exchange chromatography and von Willebrand factor (vWF) can serve as prognostic biomarkers in unstable angina (UA). The study calculated the diagnostic accuracy of these biomarkers and assessed their prognostic utility. The authors classified patients into three groups: Group 1 (stable angina, *n* = 22), Group 2 (UA without event, *n* = 71), and Group 3 (UA with event, *n* = 10), and they found that a marked elevation in plasma serotonin (>21.575 μg/mL is strongly associated with a very high short-term risk of progressing to myocardial infarction. Both biomarkers showed excellent discriminatory power (AUC = 0.975) and high positive likelihood ratios, suggesting they could be valuable tools for early risk stratification and management. (Figure 3) [29]. However, the small sample size of the event group (*n* = 10) and the lack of external validation limit the generalizability of these findings.

Figure 3, derived from the findings of Tyravska et al. [29], illustrates the diagnostic potential of Serotonin as a biomarker, highlighting its high sensitivity, preserved specificity, and a high positive likelihood ratio. Plasma serotonin demonstrated a sensitivity of 80.0% and a specificity of 95.8% for identifying patients with unstable angina (UA) at risk of short-term progression to myocardial infarction (MI), corresponding to a positive likelihood ratio (LR+) of 19.0. In comparison, von Willebrand factor exhibited a lower sensitivity of 50.0%, albeit with a slightly higher specificity of 97.2% and an LR+ of 18.0 [29].

These findings indicate that both biomarkers may have potential diagnostic value for adverse cardiovascular outcomes; however, serotonin provided superior sensitivity, thereby enhancing early detection [29]. This combination of diagnostic characteristics supports its potential utility in the timely identification and risk stratification of patients with unstable angina who are at increased risk of progression to myocardial infarction.

Importantly, these observations are consistent with earlier work by Hara et al. [30], who demonstrated that an increased plasma-to-whole-blood serotonin ratio is significantly elevated in patients with atherosclerotic disease, including those with unstable angina, reflecting increased platelet activation and vascular damage. Collectively, these data reinforce the concept that plasma serotonin functions as a dynamic biomarker of platelet activation and thrombo-inflammatory activity, with promising applicability in the risk assessment and clinical management of acute coronary syndromes.

### Major Discrepancies in Reported Serotonin Cut-Off Values

The plasma serotonin threshold reported by Tyravska et al. [29] (>21,575 ng/mL) is quite different than the cut-off identified by Odaka et al. [23] (1.68 ng/mL) for coronary microvascular dysfunction. This marked discrepancy likely reflects fundamental differences in sample processing (platelet-poor plasma vs. standard plasma), assay calibration (HPLC vs. ELISA), and patient populations (acute unstable angina vs. stable suspected angina). This heterogeneity in reported values makes clinical standardization difficult and underscores the need for assay standardization, matrix-specific reference intervals, and harmonized pre-analytical protocols before serotonin can be considered for clinical application.

## 7. Serotonin as an Emerging Biomarker

To provide a comprehensive overview of serotonin’s cardiovascular effects, Table 3 summarizes the main receptor subtypes, target cells, signaling mechanisms, and associated cardiovascular diseases discussed in this review.

Traditional biomarkers in cardiology, such as troponins, C-reactive protein, and Nt-ProBNP, are well validated, and each plays a role in the diagnosis of cardiovascular conditions. Table 4 summarizes important evidence about serotonin value alone and its comparison with other validated biomarkers.

**Figure 4:**
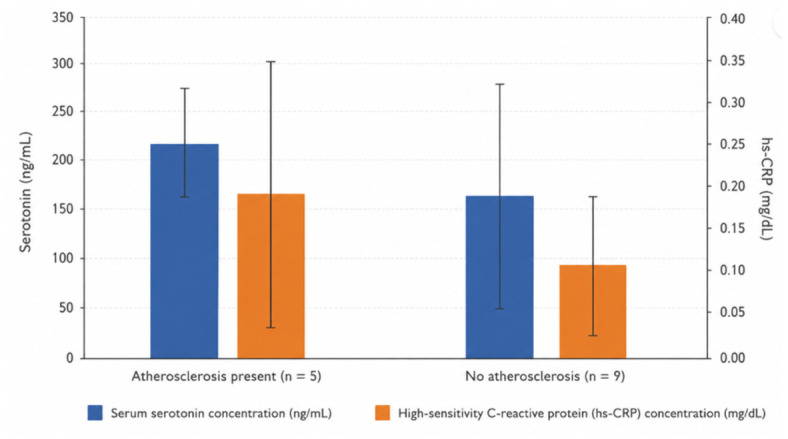
Serum serotonin (ng/mL) and hs-CRP (mg/dL) levels in groups with (*n* = 5) and without (*n* = 9) atherosclerosis. Blue bars represent serum serotonin, and orange bars represent hs-CRP. Participants with atherosclerosis were identified by pulse wave velocity (PWV) assessment adjusted for age and sex. Among these, 4/5 (80%) exhibited serum serotonin levels > 200 ng/mL and hs-CRP > 0.04 mg/dL. Error bars indicate ± SD. Among the high-risk group, 4/5 (80%) had confirmed atherosclerosis (ABI/PWV). A significant correlation was observed between both biomarkers (r = 0.271, *p* < 0.05). Data from Isobe et al. [35].

C-reactive protein (CRP) is an acute-phase reactant that is important in nonspecific host defense. High-sensitivity CRP (hsCRP) is an established inflammatory marker across a wide range of medical conditions, including atherosclerosis [35]. The study investigated hs-CRP and serum serotonin together to predict atherosclerosis in 70 apparently healthy subjects (19 men, 51 women; age range 21–68 years) and found a significant linear correlation between hs-CRP and serum serotonin (Figure 4) [35].

As shown in Figure 4, the combination of serum serotonin and hs-CRP improves the identification of individuals at increased atherosclerotic risk. A significant linear correlation was observed between serotonin and hs-CRP levels in the entire cohort (r = 0.271, *p* < 0.05). The correlation supports the interplay between inflammatory pathways and serotonergic signaling, highlighting their potential synergistic role in early disease detection and risk stratification beyond conventional clinical markers. Odaka et al. (2017) [23] found that hs-CRP levels were comparable between patients with CMD and those without CMD, whereas serum serotonin concentrations were significantly elevated in patients with CMD, suggesting that serum serotonin is more closely related to CMD than to low-grade inflammation and that plasma concentration of serotonin may be a novel biomarker for CMD in patients with angina and unobstructive coronary arteries [23].

High-sensitivity cardiac troponins (hs-cTn) are the established gold standard for diagnosing acute myocardial infarction, with high sensitivity for cardiomyocyte necrosis [33,35]. Because troponins are released primarily after irreversible myocardial injury, they provide limited information during the earlier “pre-necrotic” phase of plaque destabilization [36,37]. Serotonin’s functions and implications in the cardiovascular system suggest it may have potential as a “rule-in” marker for the progression of unstable angina toward infarction before troponins become positive. More than just a replacement for other validated biomarkers, serotonin could provide complementary information that other biomarkers have not offered until now.

Mechanistic insights from preclinical studies support the role of serotonin in atherosclerosis. Ma Y et al. 2022 [31] investigated the role of 5-HT in the development of atherosclerosis using high-fat diet-fed ApoE mice, THP-1 cell-derived macrophages, and HUVECs, where they found that co-treatment with a 5-HT synthesis inhibitor and a 5-HT receptor antagonist significantly reduced atherosclerotic plaque formation and macrophage infiltration in ApoE mice; also, 5-HT receptor activation promotes foam cell formation by enhancing triglyceride synthesis and oxidized LDL uptake via PKCε signaling, leading to lipid droplet accumulation and increased intracellular 5-HT synthesis and its mitochondrial degradation by monoamine oxidase-A (MAO-A) contribute to reactive oxygen species (ROS) production, triggering NF-κB activation and the release of pro-inflammatory cytokines such as TNF-α, IL-1β, and MCP-1. These key findings demonstrate that the pathogenesis of lipid-induced atherosclerosis is associated with activation of intracellular 5-HT2AR, 5-HT synthesis, and 5-HT degradation [31].

**Figure 5:**
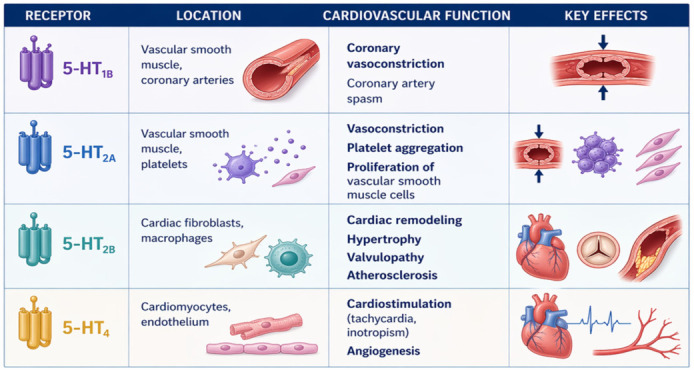
Serotonin’s receptors and their effects on the cardiovascular system. Source: own elaboration based on [5,8,11,13,31].

The implication of serotonin in the atherosclerotic process is clear. But there is a long way to validate it as a cardiac biomarker, starting with specifying exactly how to measure it, when to measure it, and what the specific message from its value is. Although interest in serotonin and its implications for cardiovascular systems has increased in recent years, more studies are needed to validate its status as a cardiac biomarker. Figure 5 shows a summary of serotonin’s receptors and their effects on the cardiovascular system.

As shown in Figure 5, 5-HT also contributes to valvopathy development. Serotonin released by platelets under high shear stress conditions, as occurs when blood passes through an abnormal valve, activates 5-HT_2_B receptors on valvular interstitial cells, stimulating their proliferation and contributing to progressive valvular remodeling. This mechanism has been implicated in the progression of rheumatic valve disease and degenerative aortic stenosis [5].

## 8. Serotonin and Precision Medicine: Challenges and Future Directions

Existing clinical guidelines recommend high-sensitivity cardiac troponins as the gold standard for diagnosing myocardial infarction, while highlighting the need for additional biomarkers to improve early risk stratification [32,38]. Considering the results of the studies discussed and the potential role of serotonin as an investigational cardiovascular diagnostic tool in precision medicine, plasma serotonin could serve as a complement for diagnosing patients with unstable angina who are at imminent risk of progressing to myocardial infarction before troponin elevation occurs [39].

Biologically, these findings are supported by the well-established roles of serotonin in the cardiovascular system and atherosclerosis [32,39]. From a precision medicine perspective, serotonin could help stratify unstable angina patients into different risk trajectories, potentially guiding earlier and more intensive interventions in those with high-risk phenotypes. Large-scale multi-omics analyses have supported the potential of serotonin as a predictor of cardiovascular risk. A recent cross-sectional multi-omics analysis of 3142 adults from the Cooperative Health Research in South Tyrol (CHRIS) study identified circulating serotonin as a novel predictor of cardiovascular and overall morbidity, independent of age and sex, with significant associations observed across cardiovascular and cardiometabolic domains. These findings suggest that serotonin’s utility may extend beyond acute coronary syndromes to improve cardiovascular risk stratification in asymptomatic or stable populations [40,41].

Despite this, several challenges remain before serotonin can be integrated into routine clinical practice, including the lack of standardized measurement protocols, the need for external validation in large multicenter cohorts, and limited evidence on whether serotonin-guided strategies improve clinical outcomes. Also, the optimal cut-off value may vary across clinical contexts. While Odaka et al. [23] identified a cut-off of 9.55 nmol/L (approximately 1.68 ng/mL) for coronary microvascular dysfunction, Tyravska et al. [29] found a much higher cut-off of >21.575 μg/mL (approximately 21,575 ng/mL) for predicting progression from unstable angina to myocardial infarction [23,29]. This divergence may reflect differences in patient populations, sample processing, or analytical methods. Tyravska measured serotonin in blood plasma, while others have used serum or platelet-poor plasma, emphasizing the need for standardization.

Future research should focus on establishing harmonized reference intervals, validating optimal cut-off values across diverse populations, standardizing analytical methods, and integrating serotonin into multivariate risk prediction models.

### TPH1 Inhibition as a Therapeutic Frontier

Pulmonary hypertension (PH) is a progressive cardiovascular disease that may appear as a primary disease, such as pulmonary arterial hypertension (PAH), or develop secondary to a varied range of cardiovascular and systemic conditions, particularly left heart disease. Regardless of etiology, PH is associated with increased morbidity and mortality, reduced functional capacity, and impaired quality of life. While specific therapies are available to treat some groups of pulmonary arterial hypertension (PAH), there are limited therapeutic options for several PH subgroups [41].

Serotonin synthesis in vascular remodeling, and specifically in PH, has also been explored and demonstrated in preclinical studies. Legchenko et al. developed TPT-001, a highly selective oral tryptophan hydroxylase-1 inhibitor TPH1 inhibitor, and tested it in the Sugen/hypoxia (SuHx) rat model of severe pulmonary arterial hypertension (PAH); demonstrating that pharmacological inhibition of peripheral serotonin synthesis with the novel TPT-001 noticeably reduced right ventricular systolic pressure (41 ± 2.3 vs. 86 ± 6.5 mmHg; *p* < 0.001), improved right ventricular function, attenuated pulmonary vascular remodeling, and suppressed perivascular infiltration of proinflammatory macrophages, CD3+ T cells, and proliferating epithelial cells [42].

Moreover, lung mRNA sequencing revealed reversal of gene networks related to smooth vascular muscle cell proliferation, reactive oxygen species production, and inflammation. These findings provided in vivo pharmacological evidence that blocking peripheral serotonin synthesis can reverse established vascular remodeling and its associated proinflammatory-proliferative gene programs, highlighting TPH1 as a possible These findings highlight the potential of therapeutic targets inhibiting peripheral serotonin synthesis via tryptophan hydroxylase 1 (TPH1) in reversing established cardiovascular pathology [42].

In summary, serotonin remains an investigational biomarker. Its inclusion alongside established markers in published studies reflects standard exploratory practice, benchmarking an emerging candidate against validated biomarkers rather than any claim of independent clinical utility. Accordingly, it could serve as a potential adjunctive biomarker for precision risk stratification in acute coronary syndromes, not replacing but, for the moment, complementing established, well-known cardiac biomarkers. Validated serotonin levels could strengthen multi-marker precision-medicine approaches for acute coronary syndrome management.

## 9. Conclusions

Serotonin plays a multifaceted role in cardiovascular physiology, particularly in endothelial function, vascular tone, and remodeling processes. The evidence supports its role as a modulator in atherosclerosis, thrombosis, and reperfusion injury.

The potential role of serotonin as a biomarker is biologically plausible given its cardiovascular effects. However, the current evidence base is limited by small study populations, important heterogeneity in serotonin measurement methods, and reported cut-off values. Despite these limitations, serotonin is an investigational biomarker that may offer a prognostic window for intervention during the “smoldering” phase of plaque instability, before irreversible myocardial injury occurs. The future of cardiovascular risk assessment likely lies in integrating new biomarkers, such as serotonin, with other thrombo-inflammatory markers into multi-marker predictive models. More than a replacement for other validated biomarkers, serotonin may represent an emerging complementary cardiac biomarker that may improve diagnostic accuracy and risk stratification in patients with non-obstructive coronary syndromes.

Although serotonin is not yet established in clinical guidelines as a routine biomarker, its mechanistic involvement in plaque instability and microvascular dysfunction makes it a strong candidate for future prospective studies.

Serotonin is an investigational biomarker with biological plausibility and growing evidence supporting its clinical utility. Its future role likely lies not in isolation, but as part of a precision cardiology approach that integrates multiple pathways involved in atherothrombosis. For the moment, its translation into clinical practice will require further validation in well-designed prospective studies.

## Figures and Tables

**Table 1:** Expression of 5-HT2A/5-HT2B receptors (According to the data in [10]).

Parameter Analyzed	Main Group (Ldlr^+/−^ Mice)	Control Group (Normal Mice)
Atherosclerotic Changes	Present. Early signs (pre-lipid stage and lipoidosis) were detected in the aorta, confirmed by Sudan III staining.	Absent. No atherosclerotic changes were detected.
Lipid Content in Heart	Increased. Morphological changes in the aorta correlated with increased lipid content in the left ventricle.	Not reported (implied to be normal).

**Table 2:** Experimental Models of Serotonin in Atherosclerosis (According to the data in [11]).

Model	Intervention	Main Outcome	Source
Ldlr^+/−^ mice	Observational expression study	Increased expression of SERT and 5-HT2A/5-HT2B receptors in aorta and myocardium associated with early atherosclerotic changes	Sadykova et al., 2025 [11]
Rat aortic rings (ex vivo)	5-HT2A antagonist (AZ928, ketanserin)	Abolition of serotonin-induced contraction	Marcinkowska et al., 2022 [13]
Human platelet-rich plasma	5-HT2A antagonists	Inhibition of platelet aggregation induced by serotonin, ADP, and collagen	Marcinkowska et al., 2022 [13]

**Table 3:** Serotonin-Mediated Mechanisms in Cardiovascular Diseases.

CVD Condition	Receptor(s)	Target Cells	Signaling Mechanism	Key Cardiovascular Effect	Refs.
Atherosclerosis	5-HT2A, 5-HT2B	VSMCs, macrophages, platelets	PLC→IP3/DAG→PKC; NF-κB activation; MAO-A→mtROS	VSMC proliferation and migration, foam cell formation, plaque progression	[8,11,13,31]
Vasospasm	5-HT1B, 5-HT2A	VSMCs, coronary endothelium	Vasoconstriction via PKC; eNOS inhibition	Coronary spasm, endothelial dysfunction, vasospastic angina.	[8,13,32]
Coronary microvascular dysfunction (CMD)	5-HT2A	Microvascular endothelium, platelets	eNOS uncoupling; platelet-mediated vasoconstriction	Impaired vasodilation, microvascular ischemia	[21,22,23]
Acute myocardial infarction/Unstable angina	5-HT2A	Platelets, VSMCs	PLC→IP3/DAG→PKC; amplification of ADP- and collagen-induced platelet aggregation	Thrombus formation, plaque rupture triggering	[13,29,30,33]
Cardiac remodeling	5-HT2B, 5-HT4	Cardiac fibroblasts, cardiomyocytes	Gs→cAMP→PKA; profibrotic and hypertrophic signaling pathways	Myocardial hypertrophy, fibrosis, arrhythmogenesis, and altered contractility	[5,34]
Valvular heart disease	5-HT2B	Valvular interstitial cells, macrophages	NF-κB activation; profibrotic signaling	Progressive valve remodeling, leaflet thickening, and fibrosis	[5]
Arterial hypertension	5-HT2A, 5-HT2B	VSMCs (conduit and resistance arteries)	Enhanced serotonergic vasoconstrictor signaling; PKC activation	Increased vascular resistance and elevated blood pressure	[4,8]
Immune-mediated inflammation	5-HT2A, 5-HT2B, 5-HT7	Macrophages, monocytes, T lymphocytes	NF-κB activation; cytokine signaling; macrophage polarization	Macrophage activation, monocyte recruitment, vascular inflammation, and plaque progression	[16,34]

**Table 4:** Summary of Key Population Studies on Serotonin as a Cardiovascular Biomarker.

Aims	Study Population	Key Findings on Serotonin	Ref. No.
To examine the clinical significance of plasma serotonin as a biomarker for coronary microvascular dysfunction (CMD) in patients with suspected angina but no obstructive coronary artery disease.	217 patients with suspected angina and unobstructive coronary arteries.	Serotonin was identified as a predictor of atherosclerosis. Plasma serotonin levels were significantly higher in patients with CMD compared to those without.	[23]
To examine whether high-sensitivity C-reactive protein (hs-CRP) and plasma serotonin can predict the presence of atherosclerosis in apparently healthy subjects	72 apparently healthy subjects (no history of CVD or related medications)	Plasma serotonin levels were significantly higher in subjects with atherosclerosis (detected by ultrasound) compared to those without. Serotonin was identified as a predictor of atherosclerosis.	[35]
To investigate the relationship between plasma serotonin (p-Ser), whole-blood serotonin (wb-Ser), and the p-Ser/wb-Ser ratio with atherosclerotic cardiovascular disease (CVD).	57 CVD patients and 64 healthy controls.	The p-Ser/wb-Ser ratio was significantly higher in CVD patients than in controls and was identified as a novel, independent marker of atherosclerotic CVD. Serotonin was identified as a predictor of atherosclerosis and CMD. Plasma serotonin levels were significantly higher in the unstable angina (UA) group than in the stable angina (SA) group and were highest in the acute myocardial infarction (AMI) group, suggesting that it is a marker of disease progression.	[33]

## Data Availability

No new data were created or analyzed in this study.

## Abbreviations

5-HIAA	5-hydroxyindoleacetic acid
5-HT	Serotonin (5-hydroxytryptamine)
ABI	Ankle-brachial index
Ach	Acetylcholine
AUC	Area under the curve
CAD	Coronary artery disease
CMD	Coronary microvascular dysfunction
CRP	C-reactive protein
ECs	Endothelial Cells
eNOS	Endothelial Nitric Oxide Synthase
hs-cTn	High-sensitivity cardiac troponins
IL-6	Interleukin-6
IPH	Intraplaque hemorrhage
LDL	Low-density lipoprotein
Ldlr^+/−^	heterozygous LDL-receptor-deficient
LRNC	lipid-rich necrotic core
MAO-A	monoamine oxidase A
MI	myocardial infarction
mtROS	Mitocondrial ROS
NF-κB	Nuclear Factor kappa-B
NO	nitric oxide
PWV	pulse wave velocity
ROS	reactive oxygen species
SERT	serotonin transporter
TCFA	thin-cap fibroatheroma
TNF-α	Tumor Necrosis Factor-α
UA	unstable angina
VEGF	vascular endothelial growth factor
VSMCs	Vascular smooth muscle cells
vWF	Willebrand factor
WHO	World Health Organization
CVD	cardiovascular disease
hsCRP	high-sensitivity CRP
